# Gene Structure Analysis of Chemokines and Their Receptors in Allotetraploid Frog, *Xenopus laevis*

**DOI:** 10.3389/fgene.2021.787979

**Published:** 2022-01-20

**Authors:** Akimasa Fukui, Masatoshi Matsunami

**Affiliations:** ^1^ Department of Biological Sciences, Faculty of Science and Engineering, Chuo University, Bunkyo-ku, Tokyo, Japan; ^2^ Graduate School of Medicine, University of the Ryukyus, Nishihara, Japan

**Keywords:** chemokine, homeolog, genome duplication, allotetraploid, subfunctionalization, neofunctionalization, *Xenopus*, amphibian

## Abstract

Chemokines, relatively small secreted proteins, are involved in cell migration and function in various biological events, including immunity, morphogenesis, and disease. Due to their nature, chemokines tend to be a target of hijacking of immunity by virus and therefore show an exceptionally high mutation rate. *Xenopus laevis* is considered an excellent model to investigate the effect of whole-genome duplication for gene family evolution. Because its allotetraploidization occurred around 17–18 million years ago, ancestral subgenomes L and S were well conserved. Based on the gene model of human and diploid frog *Xenopus tropicalis*, we identified 52 chemokine genes and 26 chemokine receptors in *X. laevis*. The retention rate of the gene in the *X. laevis* L and S subgenomes was 96% (45/47) and 68% (32/47), respectively. We conducted molecular phylogenetic analysis and found clear orthologies in all receptor genes but not in the ligand genes, suggesting rapid divergences of the ligand. *dN/dS* calculation demonstrated that *dN/dS* ratio greater than one was observed in the four ligand genes, *cxcl8b.1.S*, *cxcl18.S*, *ccl21.S*, and *xcl1.L*, but nothing in receptor genes. These results revealed that the whole-genome duplication promotes diversification of chemokine ligands in *X. laevis* while conserving the genes necessary for homeostasis, suggesting that selective pressure also supports a rapid divergence of the chemokines in amphibians.

## Introduction

Polyploidization *via* whole-genome duplication (WGD) is considered a driving force of evolutionary diversification by providing new functions through genetic redundancy (Ohno, 1970; Van de Peer et al., 2009). In general, since duplicated genes have redundant functions, one of the genes degenerates to a pseudogene (or completely lost from the genome). However, duplicated genes generated by WGD show relatively high retention rates compared to duplicated genes generated by usual tandem duplications. Although this feature is explained by proposed modes, such as the duplication-degeneration-complementation (DDC) model (Force et al., 1999) or the gene balance hypothesis (Papp et al., 2003), a complete picture of evolution after WGD is still obscure.

The African clawed frog *Xenopus laevis* is an excellent model species to infer the evolution after WGD. They have been thought to have undergone tetraploidization around 18 million years ago (Mya) by interspecific hybridization of diploid ancestors (Session et al., 2016). In contrast with the closely related diploid species, *Xenopus tropicalis*, allotetraploid species *X. laevis* has two subgenomes, L and S (Hellsten et al., 2007; Session et al., 2016). The corresponding chromosomes of *X. laevis* L and S to *X. tropicalis* have identical numbers, except for the fused 9–10 chromosome (Matsuda et al., 2015; Session et al., 2016). The homologous genes in each subgenome are defined as homeologs, discerned by suffix .L or .S. Corresponding to protein-coding genes of *X. tropicalis*, *X. laevis* holds 88 and 66% retention rates in the L and S subgenomes, respectively, and 56% of the homeologous gene pairs (Session et al., 2016). The homology of chromosomes between *X. laevis* and *X. tropicalis* was well conserved.

Chemokines are low molecular weight cytokines that regulate cell migration through activating the G-protein coupled receptors. The importance of chemokines has been more recognized as they are involved in inflammatory and homeostatic functions, including recruiting leucocytes, cell-homing, neurogenesis, angiogenesis, and regeneration (reviewed in DeVries et al., 1999; Belperio et al., 2000; Cho and Miller, 2002; Bianchi and Mezzapelle, 2020). Depending on the sequence of the two closest cysteines in the peptide, chemokines are classified into four groups: CC, CXC, CX3C, and XC (Moser et al., 2004). Chemokines are not identified in chordates, whereas teleost fish has a broad range of numbers of chemokines, 89 in zebrafish to 20 in *Tetraodon* (Nomiyama et al., 2013). Chemokine receptors are also classified into four groups: CCR, CXCR, XCR, and CX3CR, according to the biding subfamily of chemokine ligands. Chemokine receptors often have binding promiscuity that the receptor binds more than one chemokine, while a single chemokine often binds to more than one receptor (Rossi and Zlotnik, 2000; Nomiyama et al., 2011). CX3C-type chemokine ligand and receptor families have not been identified in *X. tropicalis* and teleost (Nomiyama et al., 2013).

At least 48 chemokines have been identified in the human genome, but naturally, not all species conserved the orhologies (Zlotnik et al., 2006). For example, *CXCL8* counterpart does not exist in mice (Zlotnik et al., 2006). Amphibians share the last common ancestor with mammals about 360 mya (Kumar and Hedges, 1998). Previous systemic screening exhibited 28 chemokine ligands in *X. tropicalis*, and they have no significant homology with those of mammalians, except for *cxcl12* and *cxcl14* (DeVries et al., 2006; Nomiyama et al., 2013). *cxcl12* plays essential homeostatic functions with its receptors, *cxcr4* and *ackr3* (*cxcr7*) (Lataillade et al., 2004; Burns et al., 2006; Ratajczak et al., 2006; Ratajczak et al., 2012; Hattermann and Mentlein, 2013; Puchert and Engele, 2014). In *Xenopus*, expression and function of *cxcl12* were well examined in early development, including gastrulation, germ cell migration, neural crest migration, and somitogenesis (Moepps et al., 2000; Braun et al., 2002; Fukui et al., 2007; Takeuchi et al., 2010; Mishra et al., 2013; Leal et al., 2014; Shellard and Mayor, 2016).

Chemokines can be good targets for gene evolution because they are thought to evolve relatively quickly due to competition with pathogens such as viruses and bacteria (Murphy, 2001). Although several sequences were obtained in *X. laevis* (Moepps et al., 2000; Braun et al., 2002; DeVries et al., 2006; Fukui et al., 2007; Cui et al., 2011; Goto et al., 2013; Mishra et al., 2013), elucidating the evolution of *Xenopus* chemokines will entail the whole aspect of ligands and receptors. Here, based on the latest genomic data of *X. laevis* and *X. tropicalis*, we identified all the *Xenopus* chemokine ligands and their receptors.

## Materials and Methods

### Gene Identification, Syntenic Analysis, and Phylogenetic Analysis

All identified genes were screened from gene models of the *X. laevis* annotation gene model v1.8 and v9.2 and genome assembly v9.1 and v9.2 and the *X. tropicalis* annotation v9.0 and genome assembly v9 deposit in Xenbase (www.xenbase.org), with BLAST and BLAT using known *X. tropicalis* and human nucleotide and peptide sequences as queries, following secondary screening by the obtained sequences. Gene model sequence errors were corrected manually using genome assemblies in GenomeMatcher (Ohtsubo et al., 2008). Syntenic analysis was performed with genome assembly of *X. laevis* v9.2, *X. tropicalis* v10, and *H. sapiens* GRCh38. Phylogenetic trees were generated in MEGA X (Kumar et al., 2018). Two *Xenopus* species (*X. laevis* and *X. tropicalis*), chicken (*Gallus*), and human chemokine ligand and receptor genes were aligned using CLUSTAL Omega (Sievers et al., 2011) and trimmed manually. The maximum-likelihood method was performed with 1,000 bootstraps (Felsenstein, 1985). A parameter model was estimated in MEGA X and used JTT with a gamma-distributed model for chemokine ligands and JTT with a gamma-distributed and invariable model for receptors. The inference option was a nearest-neighbor-interchange method on a neighbor-joining (NJ) tree (Saitou and Nei 1987).

### *dN/dS* Calculation

We analyzed molecular evolution rates by computing numbers of synonymous (*dS*) and non-synonymous (*dN*) nucleotide substitutions per site for each pair of *X. tropicalis versus X. laevis* L or *versus X. laevis* S gene. A low ratio (*dN/dS* < 1) indicates purifying selection, which maintains similarity between orhologies, whereas a high ratio (*dN/dS* > 1) indicates positive selection, promoting rapid divergence of the orhologies. The *dN/dS* ratios were calculated by the CODEML program implemented in the PAML v. 4.9j package (Yang, 2007). We used the free ratio model (model = 1, NS site = 0, fix omega = 0) for *dN/dS* calculation of each branch.

### Transcriptome Correlation Analysis

RNA-seq data analysis and transcriptome correlation are described previously (Session et al., 2016; Watanabe et al., 2017). Expression profiles of identified genes were extracted from the series of oocytes (oocyte stages I-II, III-IV, and V-VI), egg, early embryos (stages 8, 9, 10.5, 12, 15, 20, 25, 30, 35, and 40), and adult organs (brain, eye, lung, stomach, intestine, liver, pancreas, kidney, testis, ovary, heart, muscle, skin, and spleen) of *X. laevis* J-strain, analyzed by Session et al. (2016), using RNA-seq short reads deposited in NCBI Gene Expression Omnibus (accession number GSE73430 for oocytes and all embryos, GSE73419 for all adult organs). The data include biological replicates (named “Taira201203” and “Ueno201210”) for embryos and adult organs but no replicate for oocytes (only “Ueno201210”). These distinct datasets were called Clutch T and Clutch U, respectively. Transcripts per million (TPM) values of each gene in each clutch are presented in Supplementary Table S1.

Prior to transcriptome correlation analysis, all TPM values ≤ 0.5 were reduced to 0 because transcriptome data less than 0.5 TPM is considered to be irreproducible (Session et al., 2016). The transcriptomic dataset from 11 developmental stages (egg to stage 40) and 14 adult tissues were separately analyzed. Also, Clutch T and Clutch U were separately analyzed to examine the reproducibility in biological replicates. Any gene whose TPM value is ≤0.5 for all samples was removed from the analysis. Correlations of expression profiles between homeologs were examined using Pearson’s correlation and Student’s paired *t*-test on log2-transformed data [log2 (TPM+1)] as described by Berthelot et al. (2014). Homeologous pairs were categorized into four groups based on 1) correlation (HC: high correlation, *p* ≤0.05; NC: no correlation, *p* >0.05, Pearson’s correlation test) and 2) expression levels (SE: same expression levels, *p* >0.05; DE: different expression levels, *p* ≤0.05, Student’s paired *t*-test). Finally, we collected homeologous pairs which were consistently categorized into the same group in both Clutch T and Clutch U. If the category was inconsistent between Clutches, those genes were categorized as “inconsistent (inc).” Also, Clutches T and U were analyzed separately to examine reproducibility in biological replicates. Any gene with a TPM value ≤0.5 for all samples was excluded from analysis and labeled “n/a.”

## Results

### Overview of Gene Annotation and Identities of *Xenopus* Chemokine Ligand and Receptor Genes

**FIGURE 1:**
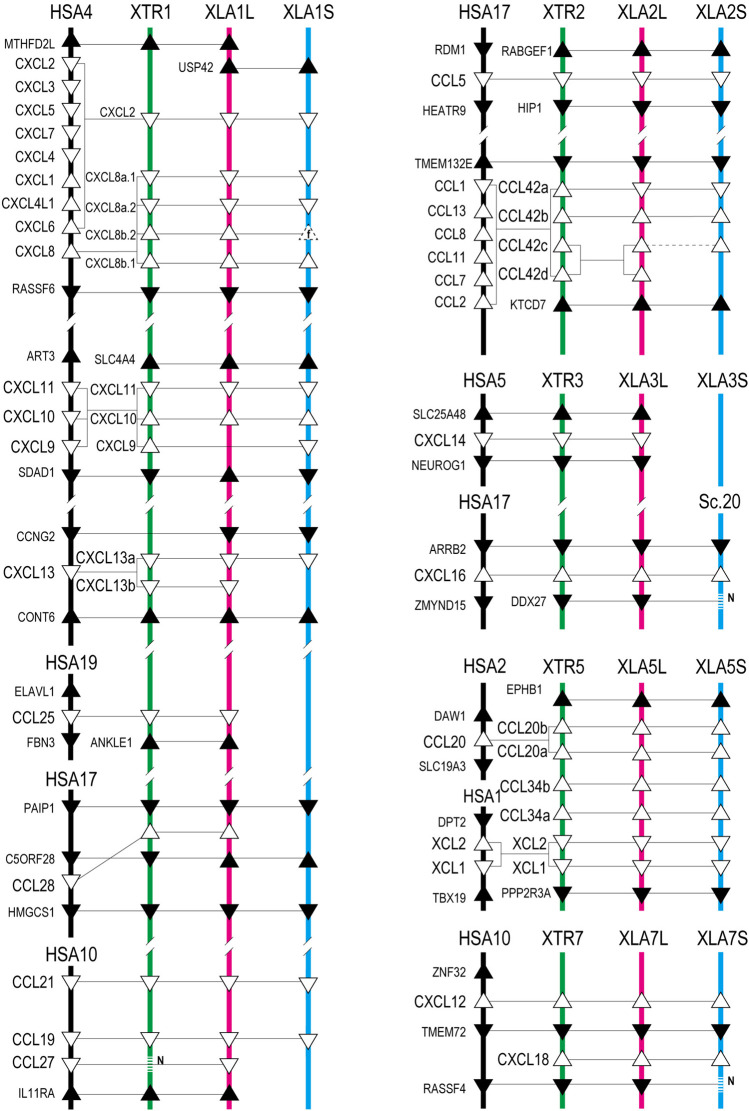
Genomic organization of *Xenopus* chemokines. Positions of chemokine genes (open triangles) and flanking genes (closed triangles) with direction are indicated in the order of *Xenopus* chromosome numbers. *Chromosomes Abbreviations*. HSA: *H. sapiens* (black lines); XTR: *X. tropicalis* (green lines); XLA_L and XLA_S: *X. laevis* L and S subgenome (red and blue lines), respectively. Sc is a scaffold number that is unbuilt in the chromosome assembly. The homologous relationship presented by connected lines was analyzed phylogenetically. The dotted line with N represents the genes unidentified with N-gap. Triangles drawn with dotted line show fossil genes (f).

**FIGURE 2:**
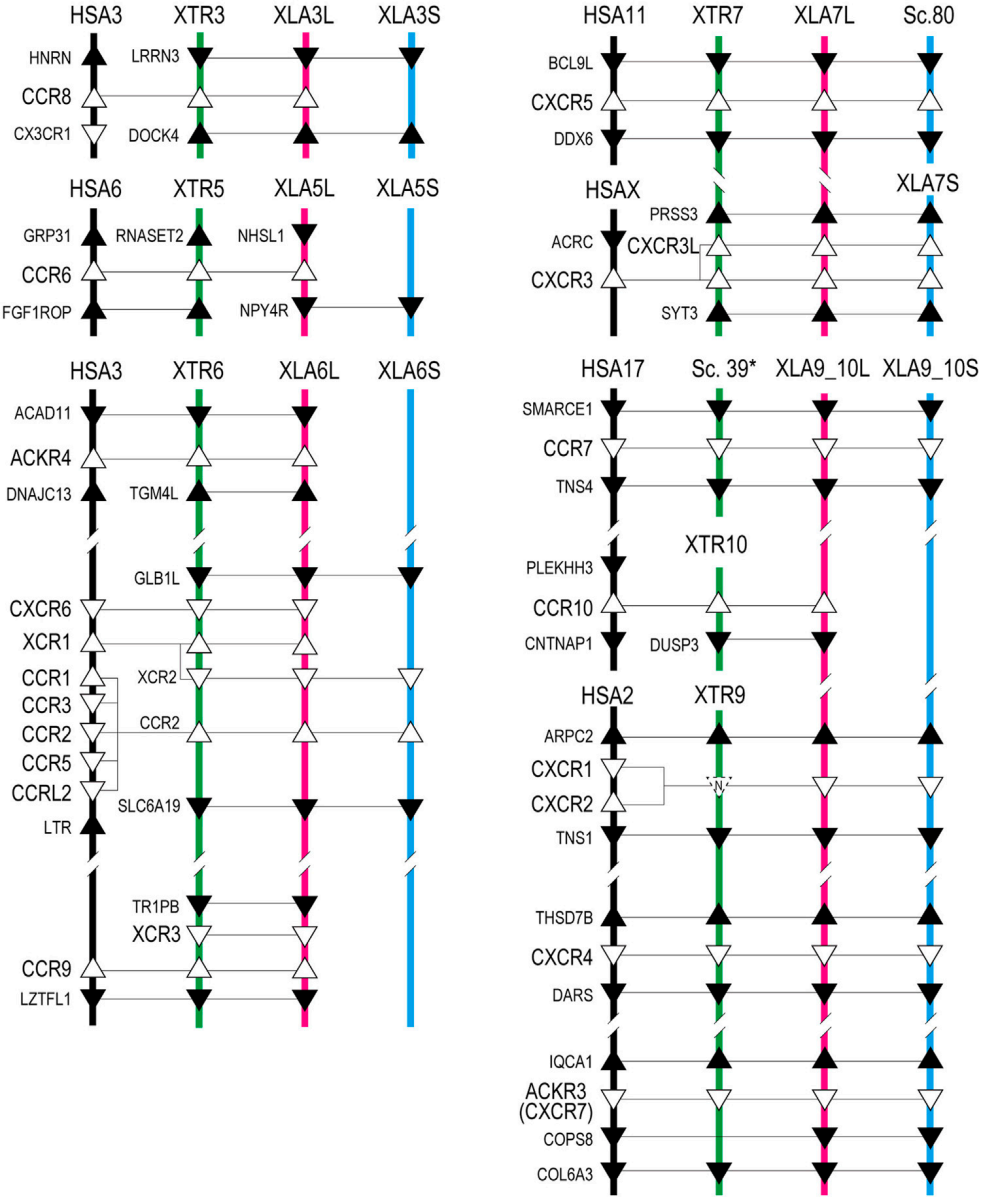
Genomic organization of *Xenopus* chemokine receptors. Representation is the same as Figure 1.

**FIGURE 3:**
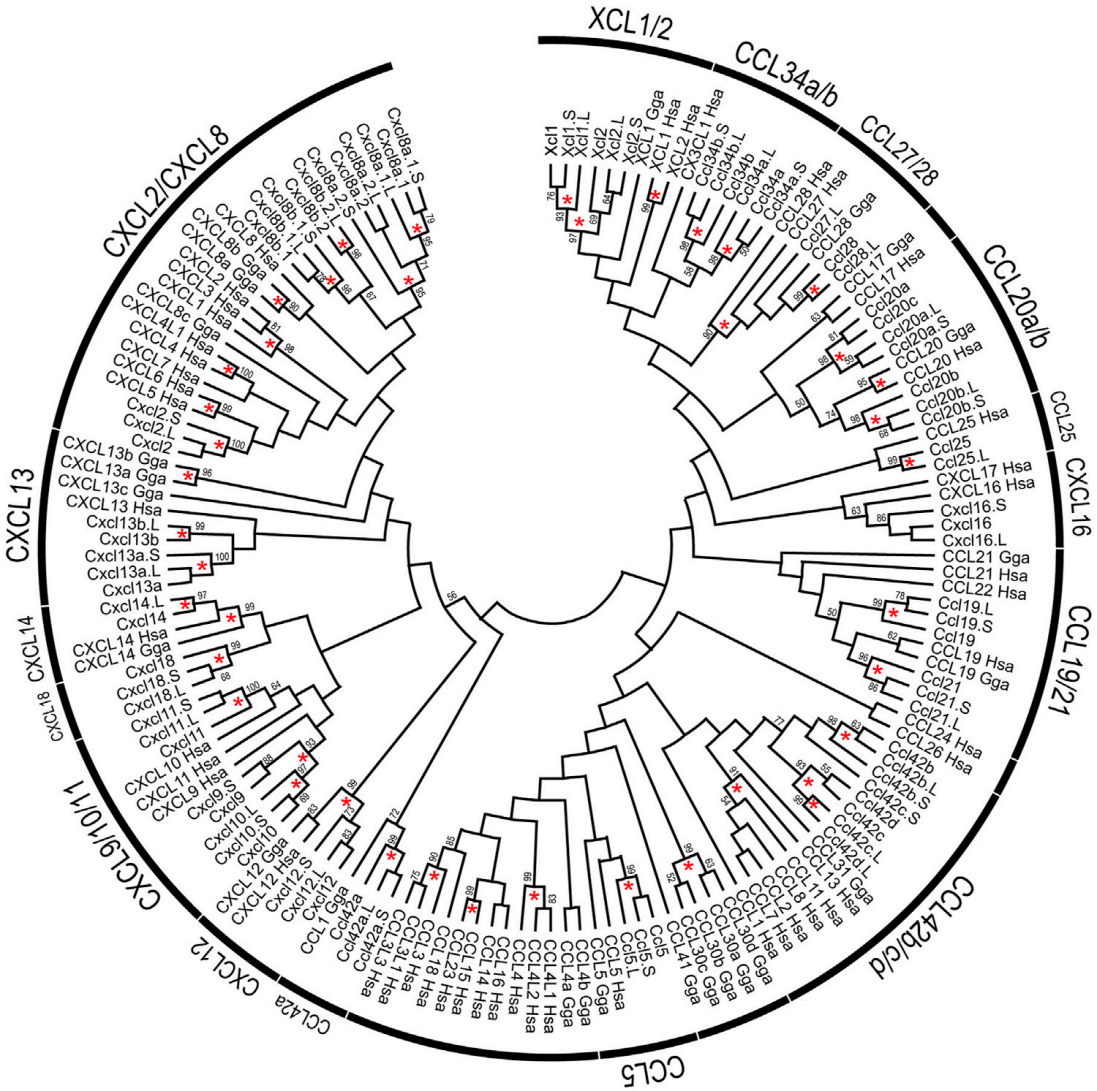
Phylogenetic tree of chemokine ligands. The tree indicates 154 chemokine proteins, including 52 of *X. laevis*, 30 of *X. tropicalis*, 24 of *G. Gallus*, and 48 of *H. sapiens* genes. Chemokine names related to *Xenopus* represented on the arcs. Bootstrap values greater than 50% were indicated, and asterisks show values greater than 90%. The alignment of chemokine proteins was prepared using CLUSTAL omega. Maximum likelihood methods using full-length were performed with 1,000 bootstraps using JTT with Gamma-distributed model, and inference option was a nearest-neighbor interchange method on NJ tree.

**FIGURE 4:**
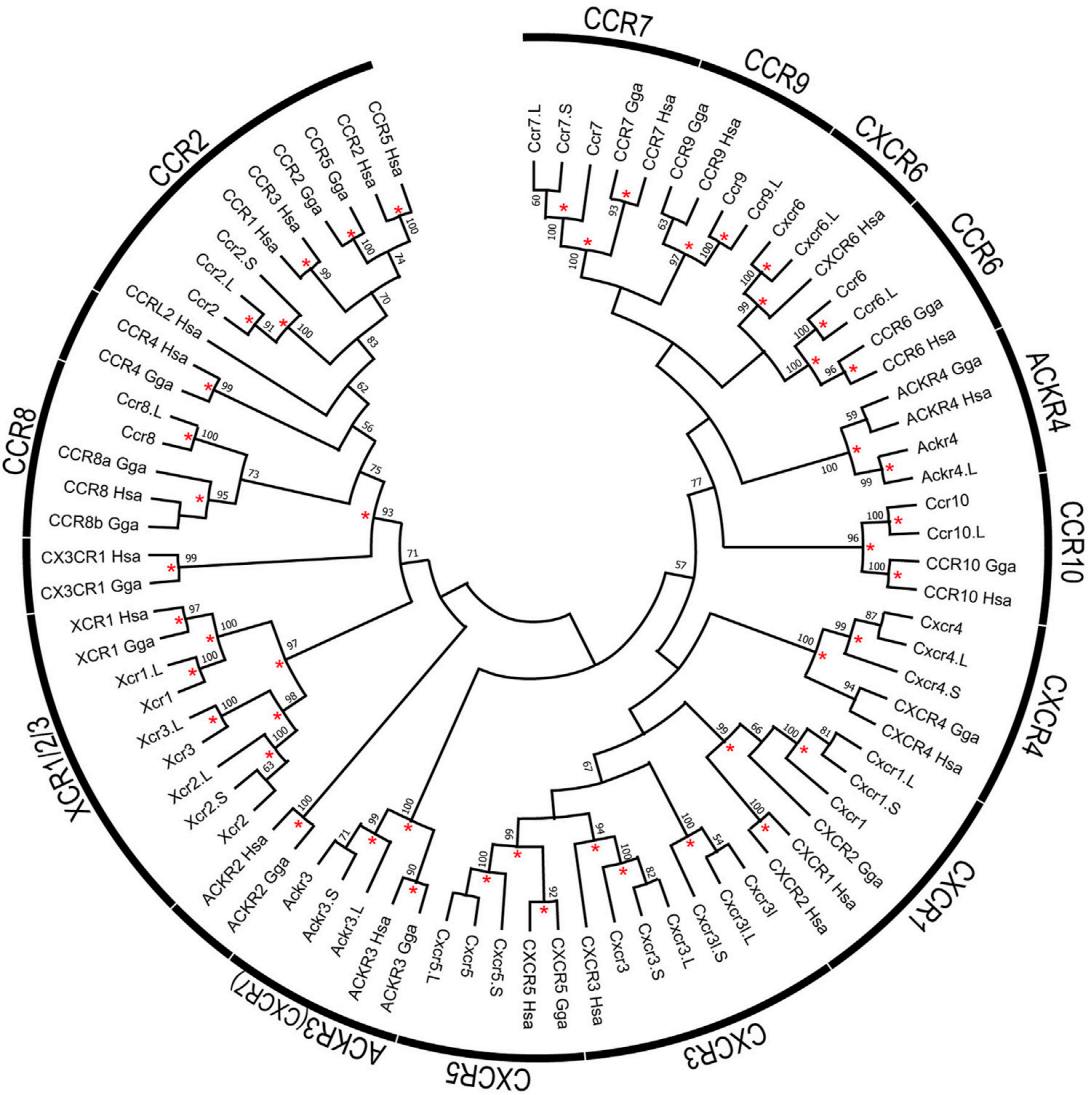
Phylogenetic tree of chemokine receptors. The tree indicates 82 chemokine receptor proteins, including 26 of *X. laevis*, 17 of *X. tropicalis*, 17 of *G. Gallus*, and 22 of *H. sapien*s genes. Receptor names represented on the arcs. Bootstrap values greater than 50% were indicated, and asterisks show values greater than 90%. The alignment of the receptor proteins was prepared using clustal omega and trimmed manually as 297 peptides with gaps. Maximum likelihood methods were performed with 1,000 bootstraps using the JTT model with Gamma distribution and invariant sites and complete deletion of gaps/missing data and inference option was a nearest-neighbor interchange method on NJ tree.

**FIGURE 5:**
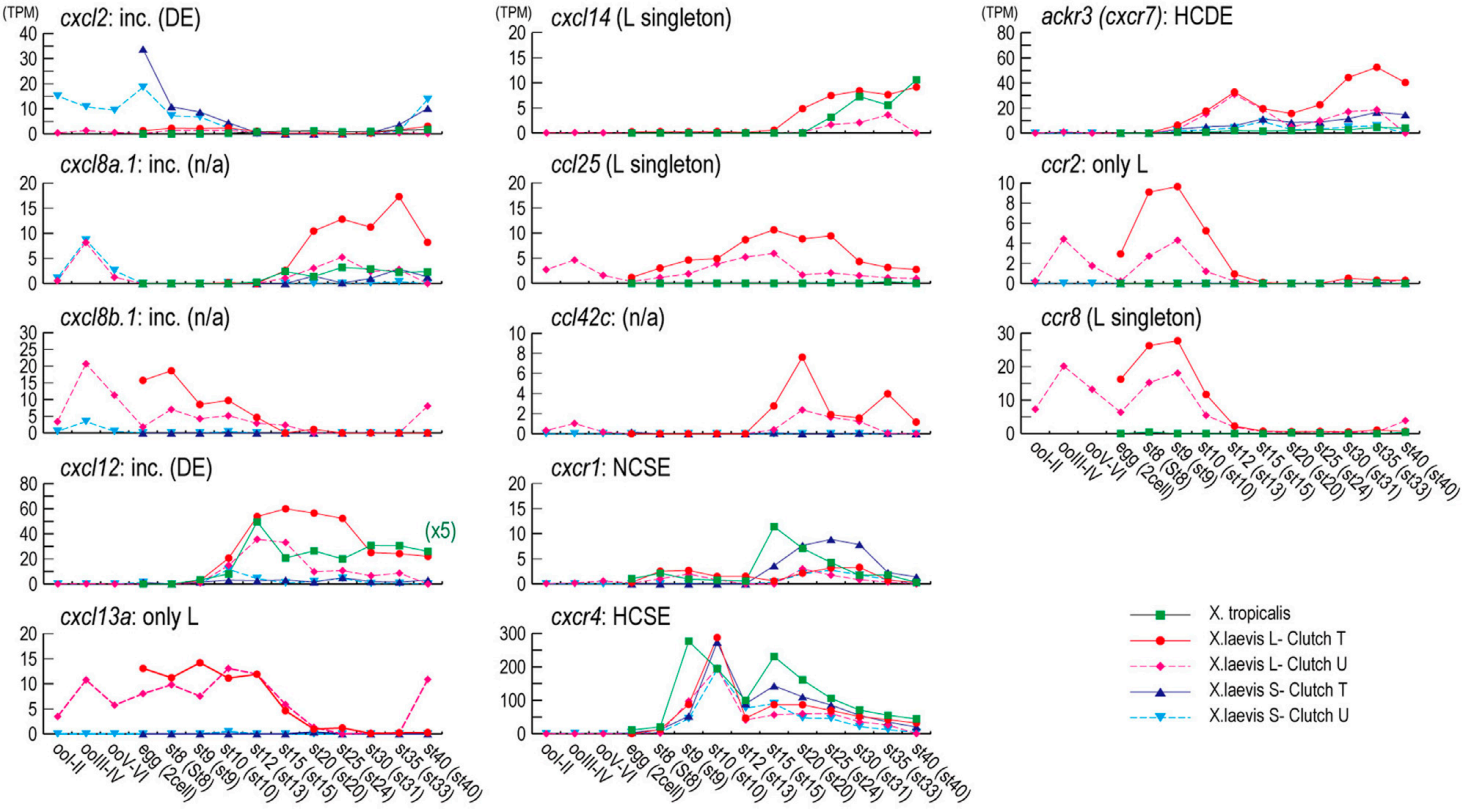
Expression profiles during oogenesis and embryogenesis. Genes with a max TMP value of 5 or higher during oogenesis and embryogenesis are presented. Expression profiles for X. *tropicalis* were obtained from Tan et al. (2013). Unfortunately, *cxcl8*, *cxcl13a*, and *ccl42c* have no expression profile of *X. tropicalis*. The vertical axis shows the expression level (TPM), and the horizontal axis is the developmental stages of *X. laevis*, *X. tropicalis* indicated within parentheses. All TPM values are shown in Supplementary Table S1. Orthologous family names and results of transcriptome correlation analysis are indicated on the upper left of each graph. *Symbols*. Square: *X. tropicalis* (green); circle,: *X. laevis* L-clutch T (red); diamond: *X. laevis* L-clutch U (magenta); triangle: *X. laevis* S-clutch T (blue); reverse triangle: *X. laevis* S-clutch U (cyan). Note that the TPM value of *X. tropicalis* in *cxcl12* was indicated one-fifth scale to increase the resolution (x5).

**FIGURE 6:**
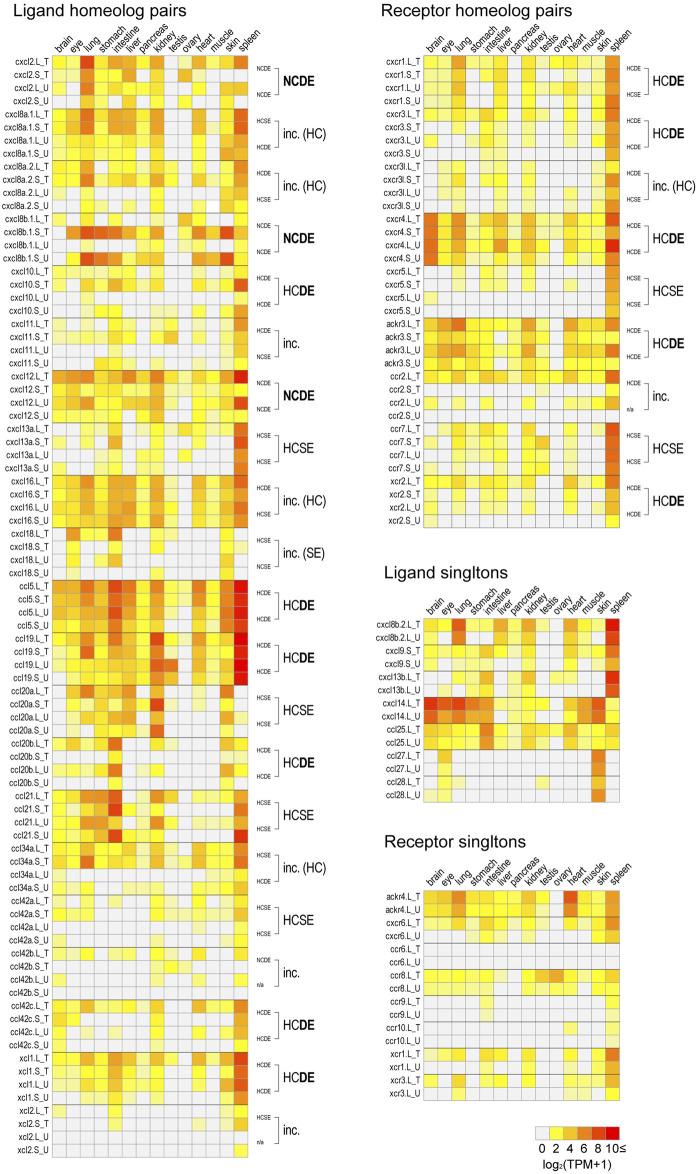
Expression profiles in adult tissues. log2 transformed TPM values in Clutch T and Clutch U [log2 (TPM+1)] of all chemokine ligand and receptor genes of brain, eye, lung, stomach, intestine, liver, pancreas, kidney, testis, ovary, heart, muscle, skin, and spleen are presented with heat maps. For each homeologous pair, transcriptome correlation groups are indicated on the right side of panels. In cases of the “inc.” group, their details are described with parentheses. If results from two clutches showed half-consistency, their common results are indicated (HC or SE). Singletons were presented in the separated panels. All TPM values are shown in Supplementary Table S1.

Based on the gene model of human and *X. tropicalis*, we screened 52 chemokine ligand genes that contained 44 homeologs (22 pairs) and 8 singletons from *X. laevis* genome assembly (Table 1, Supplementary Data S1, S2). We also reidentified 30 chemokine genes in *X. tropicalis* assemblies and represented the retention rate of the gene in *X. laevis* L subgenome as 93% (28/30) and that of S as 79% (23/30) (Figure 1). Furthermore, 26 chemokine receptors were identified in *X. laevis* genome, including 18 homeologs (9 pairs) and 8 singletons (Table 1, Figure 2, Supplementary Data S3, S4). The retention rate of the *X. tropicalis* genes in *X. laevis* L and S subgenomes was 100% (17/17) and 53% (9/17), respectively (Figure 2). The average amino acid sequence homology between homeologs was 88 and 77% for the receptors and ligands, respectively (Table 1). We conducted a molecular phylogenetic analysis using four vertebrate species (*H. sapiens, G. gallus, X. laevis*, and *X. tropicalis*). We found that all receptor genes (17/17) showed clear orthology in the phylogenic tree among species, but only 43% (13/30) of the ligand genes retained clear orthology (Figures 3, 4). Further, *dN/dS* analysis against *X. tropicalis* sequences indicated that the *dN/dS* ratio greater than one of either homeologs was found in 19% (4/21) of the ligands but not in all of the receptors (0/8) (Table 1). These findings demonstrate that the mutation rates remarkably increased in the ligand. RNAseq analysis indicated the expression of eight chemokine ligands and five receptors in embryogenesis (TPM value >5), and only *cxcl2.S* revealed S dominant expression (Figure 5). In adult tissues, L dominant expression of most genes was observed, but some showed S dominant expression described in distinct. Transcriptome correlation analysis indicated six high correlation-similar expression (HCSE), 10 high correlation-different expression (HCDE), 0 no correlation-similar expression (NCSE), and three no correlation-different expression (NCDE), with 11 inconsistent expression (inc.) in adult tissues (Table 1, Figure 6). We describe the chemokine ligands and receptors below in order of chromosome numbers.

**TABLE 1:** Review of chemokine ligands and receptors in *Xenopus laevis*. Loci were estimated by the closest locus of FISH results demonstrated in Session et al. (2016). Orthologies were obtained from molecular phylogenetic analysis and syntenic analysis. Peptide sequence homology between L and S homeologous genes was calculated by CLUSTAL omega using full-length predicted peptide. Columns of transcriptome correlation analyses show the categories of HC: high correlation; NC: no correlation; SE: same expression levels; DE: different expression levels. “inc.” indicates inconsistent categories (see Materials and Methods). Note that cxcl16, ccl5, ccl21, ccl28, ccl42a, ccl42b, ccl42c, ccl42d, xcl1, xcl2, ccr2, and ccr8 genes are unidentified in teleosts, and cxcl18, ccl34a, ccl34b, and cxcr3l genes are unidentified in mammals (Nomiyama et al., 2013).

Gene name	Loci		L/S peptide Homology (%)	*dN/dS*		Transcriptome correlation analyses		Notes
	L	S		Xtr-Xla.L	Xtr-Xla.S	Embryonic	Tissue	
Ligands								
cxcl2	1Lp12	1Sp12	80	0.43	0.42	inc. (DE)	NCDE	Maternal S dominant expression
cxcl8a.1	1Lp12	1Sp12	87	0.18	0.29	inc. (n/a)	inc. (HC)	Embryonic L dominant expression
cxcl8a.2	1Lp12	1Sp12	89	0.95	0.44	No expression	inc. (HC)	
cxcl8b.1	1Lp12	1Sp12	68	0.49	2.13	inc. (n/a)	NCDE	Maternal L dominant expression
cxcl8b.2	1Lp12	—	—	n.d.		—	—	
cxcl9		1Sp12	—		n.d.	—	—	
cxcl10	1Lp11-12	1Sp12	92	0.16	0.51	No expression	HCDE	
cxcl11	1Lp11-12	1Sp12	78	0.37	0.65	No expression	inc.	
cxcl12	7Lq11-12	7Sq11	93	0.1	0.41	inc. (DE)	NCDE	Embryonic L dominant expression
cxcl13a	1Lp11-12	1Sp12	86	0.22	0.69	(n/a)	HCSE	Maternal cxcl13a.L expression
cxcl13b	1Lp11-12	—	—	n.d.		—	—	
cxcl14	3Lq13	—	—	n.d.		—	—	Embryonic expression (L singleton)
cxcl16	3Lq34-35	Sc.20	63	0.81	0.61	No expression	inc. (HC)	Unidentified in teleosts
cxcl18	7Lq11-12	7Sq11	72	0.96	1.88	No expression	inc. (SE)	Unidentified in mammals
ccl5	2Lp13	2Sp13	89	0.58	0.6	No expression	HCDE	Unidentified in teleosts
ccl19	1Lq35	1Sq35	84	0.6	0.37	No expression	HCDE	
ccl20a	5Lq32	5Sq24-31	72	0.25	0.39	No expression	HCSE	
ccl20b	5Lq32	5Sq24-31	83	0.41	0.24	No expression	HCDE	
ccl20c*	—	—	—					No syntenic ortholog in *X. laevis*
ccl21	1Lq35	1Sq35	60	0.68	1.04	No expression	HCSE	Unidentified in teleosts
ccl25	1Lq12	—	—	n.d.		—	—	Embryonic expression (L singleton)
ccl27	1Lq35	—	—	n.d.		—	—	
ccl28	1Lq33-34	—	—	n.d.		—	—	Unidentified in teleosts
ccl34a	5Lq32	5Sq24-31	80	0.2	0.42	No expression	inc. (HC)	Unidentified in mammals
ccl34b	5Lq32	5Sq24-31		0.5	0.38	No expression	n.d.	Unidentified in mammals
ccl42a	2Lq13-14	2Sq14-15	73	0.49	0.59	No expression	HCSE	Unidentified in teleosts
ccl42b	2Lq13-14	2Sq14-15	73	0.05	0.56	No expression	inc. (n/a)	Unidentified in teleosts
ccl42c	2Lq13-14	2Sq14-15	45	0.24	0.57	(n/a)	HCDE	Embryonic L dominant expression
								Unidentified in teleosts
ccl42d	2Lq13-14	—	—	n.d.			—	Unidentified in teleosts
xcl1	5Lq32	5Sq24-31	75	1.96	0.4	No expression	HCDE	Unidentified in teleosts
xcl2	5Lq32	5Sq24-31	72	1	0.94	No expression	inc. (n/a)	Unidentified in teleosts
Receptors								
cxcr1	9/10Lq21	9/10Sq21	93	0.16	0.11	NCSE	HCDE	Embryonic S dominant expression
cxcr3	7Lq23	7Sq23	81	0.74	0.57	No expression	HCDE	
cxcr3l	7Lq23	7Sq23	89	0.22	0.39	No expression	inc. (HC)	Unidentified in mammals
cxcr4	9/10Lq24	9/10Sq21	97	0.02	0.06	HCSE	HCDE	Embryonic even expression
cxcr5	7Lq12-13	Sc.80	79	0.43	0.36	No expression	HCSE	
cxcr6	6Lp13	—	—	n.d.			—	
ackr3 (cxcr7)	9/10Lq24	9/10Sq31	97	0.07	0.06	HCDE	HCDE	Embryonic L dominant expression
ackr4 (ccrl1)	6Lp14	—	—	n.d.				
ccr2	6Lp13	6Sp12	80	0.55	0.73	(n/a)	inc. (n/a)	Maternal ccr2.L expression
								Unidentified in teleosts
ccr6	5Lq11	—	—	n.d.			—	
ccr7	9/10Lp12	9/10Sp14	87	0.34	0.3	No expression	HCSE	
ccr8	3Lq16-21	—	—	n.d.		—	—	Maternal expression (L singleton)
								Unidentified in teleosts
ccr9	6Lp12-13	—	—	n.d.		—	—	
ccr10	6Lp22	—	—	n.d.		—	—	
xcr1	6Lp13	—	—	n.d.		—	—	
xcr2	6Lp13	6Sp12	86	0.56	0.17	No expression	—	
xcr3	6Lp12-13	—	—	n.d.		—	—	

### Chemokine Ligands of *Xenopus Laevis*

A large cluster of CXC-type chemokines was found between flanking genes *rasssf6* and *usp42* in *Xenopus* chromosome 1, which contained four homeologous pairs and one L singleton (Figure 1). They have no one-to-one relationship with human orhologies indicated by molecular phylogenetic analysis (Figure 3). Among *cxcl8* homeologous genes, *cxcl8b.1* were distinct in sequence homology (68%) and expression patterns, with L dominant in embryos and S dominant in tissues (Table 1, Figures 5, 6). Since the X residue of the CXC motif has been noted for altering the binding ability to the receptor (Wedemeyer et al., 2020), we examined that, of the *cxcl8* chemokine family, four *cxcl8* homologs between *X. laevis* and *X. tropicalis* were conserved as CQC in *cxcl8b.2* and *cxcl2*, and CLC in *cxcl8a.2* and *cxcl8a.1* homologous genes. However, *cxcl8b.1* homologs presented unique sequences as CKC in *cxcl8b.1*, CRC in *cxcl8b.1.L*, and CQC in *cxcl8b.1.S*, respectively. Finally, a higher *dN/dS* ratio (2.32) of *cxcl8b.1.S* exhibited potentially positive selection or relaxation, markedly suggesting that this gene experienced unusual evolution.

*cxcl9*, *cxcl10*, and *cxcl11* exhibited the cluster on chromosome 1, and *cxcl9.S* retained the only *cxcl9.S* singleton among the chemokine ligands. They have no one-to-one relationship with human orhologies (Figure 3). Relatively S dominant expression of these genes was observed in tissues (Figure 6).

*cxcl13* paralogs showed tandem duplication in the *Xenopus* genome. Their synteny is consistent with human, located between flanking genes *ccng2* and *cont6*. Expression levels during oogenesis and embryogenesis presented L dominant expression, whereas tissue expression was highly correlated (HCSE) (Figures 5, 6).

*ccl25* had no synteny conservation between *Xenopus* and human. This gene was adjacent to *ankle1* in *Xenopus*, whereas *fbn3* and *elavl1* were in humans (Figure 1). Broad expressions were found through oogenesis and embryogenesis and among adult tissues (Figures 5, 6), suggesting homeostatic function.

*ccl28* was adjacent to the *c5orf28* in both *Xenopus* and human, but their positions were rearranged (Figure 1). *ccl28* formed a clade with *ccl27* in phylogenetic analysis (Figure 3). Both *ccl27* and *ccl28* were L singleton and expressed in the skin dominantly (Figure 6).

*ccl21*, *ccl19*, and *ccl27* formed a small cluster flanked by *1l11ra* in chromosome 1 and was observed in human. Note that *il11ra.S* was not found, probably due to gene loss in the S subgenome. Unfortunately, *ccl27* of *X. tropicalis* was unidentified with N deletion in the genome sequence. *ccl21* and *ccl19* formed a clade in phylogenetic analysis (Figure 3). *ccl19* homeologous pair revealed different expression (HCDE). *ccl19.L* was most highly expressed in testis among the chemokines examined (Figure 6). *ccl21.L* was dominantly expressed in lung, stomach, kidney, and S dominant expression in intestine and spleen (Figure 6).

*ccl5* was one of the abundant CC-type chemokines in *Xenopus* (Figure 6 and Supplementary Table S1) and exhibited HCDE in transcriptome correlation analysis. Synteny of *ccl5* was inconsistent between *Xenopus* and human as flanking genes of *ccl5* were *rabgef1* and *hip1* for *Xenopus*, but *RDM1* and *HEATR9* for human (Figure 1). Intriguingly, CCL5 can bind to three receptors, CCR1, CCR3, and CCR5 (Zlotnik et al., 2006), but all orhologies were not identified in *Xenopus*.

*ccl42a*, *ccl42b*, *ccl42c*, and *ccl42d* formed a cluster (Figure 1). The nomenclature of these genes depends on Nomiyama et al. (2013). *ccl42c* and *ccl42d* have no obvious orthology in phylogenetic analysis (Figure 3), which may be due to gene conversion or species-specific tandem duplication. Notably, the *ccl42* cluster was adjacent to *tmem132e*, as well as one of the human chemokine clusters. Further, *ccl42a* clade included *G. gallus ccl1* with the bootstrap value of 72%, and *ccl42b*, *ccl42c*, and *ccl42d* formed a clade with human *CCL1, CCL13, CCL8, CCL11, CCL7*, and *CCL2.* These findings suggest that *ccl42* chemokines were orhologies to mammalian *ccl* cluster genes. *ccl42c.L* was slightly expressed in embryogenesis, and *ccl42a.L*, *ccl42b.L*, and *ccl42c.L* were expressed in spleen L dominantly (Figures 5, 6).

*cxcl14* is one of the most conserved chemokine genes among vertebrates and has been reported as a novel ligand of *cxcr4* similar to *cxcl12* (Tanegashima et al., 2013). RNAseq analysis demonstrated that the expression was detected from neurula, and relatively higher expressions were observed in the brain, skin, lung, stomach, eye, and muscle (Figures 5, 6).

*cxcl16* is a transmembrane-type chemokine (Matloubian et al., 2000; Abel et al., 2004), and the CXC motif is replaced by the CC motif in the *Xenopus* genus. The peptide sequence homology between homeologs was relatively low (68%).

*xcl1, xcl2, ccl34a, ccl34b, ccl20a*, and *ccl20b* formed a cluster in *Xenopus* genomes, whereas human orhologies *XCL1*, *XCL2,* and *CCL20* were scattered in different chromosomes (Figure 1). Further, the flanking gene of *ccl20a* is *ppp2r3a*, whereas those of human *CCL20* are *SLC19A3* and *DAW1*. Phylogenetic analysis indicated *ccl20b*, not *ccl20a*, was relatively similar to human and bird *CCL20*. A distinct expression pattern was observed between *ccl20a* and *ccl20b* of a higher level of *ccl20a* homeologs in the liver and kidney and *ccl20b* homeologs in the stomach (Figure 6). *ccl20a.S* was dominantly expressed in the kidney. *ccl20b* homeologous pair exhibited different expression patterns (HCDE). *ccl20c* identified in *X. tropicalis* (Nomiyama et al., 2013) had no syntenic ortholog in *X. laevis*.

*ccl34a* and *ccl34b* formed a clade with *xcl1* and *xcl2* in molecular phylogenetic analysis (Figure 3). The gene name of *ccl34* depends on Nomiyama et al. (2013). *ccl34a.L* exhibited expression in the lung and spleen dominantly. There are no expression data of *cxcl34b* because this is a newly identified gene in this study after Session et al. (2016).

*xcl1* and *xcl2* were clustering genes likewise orhologies of human. However, molecular phylogenetic analysis exhibited separated branches of *Xenopus* and human orhologies (Figure 3), and the *Xenopus xcl1* and *xcl2* were separated by each gene. This no one-to-one relationship suggests species-specific tandem duplication in the *Xenopus* ancestor. Intriguingly, *dN/dS* ratio of 1.96 and 1.00 in *xcl1.L* and *xcl2.L*, respectively, indicated higher relaxation in both L homeologs. Transcriptome correlation analysis demonstrated different expression (HCDE) between *xcl1* homeologs. *xcl2.S* was expressed S dominantly in the spleen (Figure 6).

*cxcl12* and *cxcl18* were adjacent to *tmem72* and on the opposite side (Figure 1). Expression profiles of *cxcl12* homeologs demonstrated L dominant expressions in embryogenesis and adult tissues (Figures 5, 6). *cxcl18* ortholog was found in the teleost, was unidentified in mammals, and has been not yet reported function (Nomiyama et al., 2013). *cxcl18.L* was dominantly expressed in the eye and intestine. *dN/dS* ratio of 1.85 in *cxcl18.S* indicated relaxation or positive selection.

### Chemokine Receptors of *Xenopus Laevis*

*ccr8* was L singleton gene located between flanking genes *lrrn3* and *dock4* on chromosome 3, whereas the gene order was not conserved in human (Figure 1). RNAseq analysis demonstrated the unique pattern of *ccl8.L*. This gene was expressed in oocytes to blastula through embryogenesis and testis and ovary of adult tissues (Figure 6). *ccr8* is a candidate receptor for *ccl1* (Tiffany et al., 1997), and the phylogenetic analysis indicated *G. gallus ccl1* organizes a clade with *ccl42a* (Figure 3), suggesting a functional similarity between *ccl42a* and *ccl1*.

*ccr6* was located between flanking genes *lrrn3* and *dock4* in *X. tropicalis* and *nhsl1* and *npy4r* in *X. laevis* on chromosome 5, respectively (Figure 1). Partial synteny of *X. tropicalis* was conserved in human but not in *X. laevis* subgenomes. Phylogenetic analysis indicated clear orthology between species (Figure 4). Therefore, syntenic inconsistency may be due to chromosome rearrangement (Session et al., 2016). RNAseq analysis indicated no expression of *ccr6* in all tissues. Human *CCR6* is identified as a *CCL20* receptor (Baba et al., 1997; Hieshima et al., 1997).

*ackr4*, also known as *ccrl1*, is a decoy receptor that controls chemokine levels by sequestrating the ligands. *Xenopus ackr4* gene was located between *acad11* and *tgm4l* on chromosome 7 as L singleton. Locus around *ackr4* and its surrounding genes were not identified in the available S subgenome. RNAseq analysis demonstrated broad expression in adult tissues with a higher level in the heart, except for the ovary. Since human ACKR4 can bind to CCL2, CCL8, CCL13, CCL19, CCl20, CCL21, and CCL25 (Gosling et al., 2000; Schweickart et al., 2000; Matti et al., 2020), these chemokines might be the candidate ligands for *Xenopus ackr4*. Note that *ccl42b, ccl42c, ccl42d* were candidate orhologies for *ccl2*, *ccl8*, and *ccl13* (see ligands sections).

*cxcr6*, *xcr1*, *xcr2*, and *ccr2* formed a cluster in *Xenopus* chromosome 6. *cxcr6* and *xcr1* were L singleton, whereas *xcr2* and *ccr2* retained both homeologs. Flanking genes of this cluster were different between *Xenopus* and human, but synteny within-cluster was well-conserved. *xcr1* seems to be duplicated in the ancestral *Xenopus* genome. Notably, three *xcr1*-type receptors, *xcr1*, *xcr2*, and *xcr3*, existed in the *Xenopus* genome with clear orthology (Figure 2). In contrast, synteny and phylogenetic analysis demonstrated that *Xenopus ccr2* corresponds to a single ortholog for human *CCR1*, *CCR2*, *CCR3*, *CCR5*, and *CCRL2* (Figures 2, 4). RNAseq analysis of *ccr2* homeologs indicated L dominant expression in embryogenesis and adult tissues.

*ccr9* was next to *xcr3* between *tr1pb* and *lztfl1* in chromosome 6 as L singleton and weakly expressed in the lung and spleen. The candidate ligand *ccl25* existed in the *Xenopus* genome.

Human *CXCR5* is a candidate receptor for *CXCL13* and has been reported to be essential for B cell migration (Förster et al., 1996). Surrounding synteny of this gene was conserved between *Xenopus* and human. Expression of both *cxcr5.L* and *cxcr5.S* was found in the spleen.

*Xenopus cxcr3* and *cxcr3l* were tandemly aligned between *prss3* and *syt3* in chromosome 7. There was no syntenic conservation with human. Phylogenetic analysis revealed that *cxcr3*, *cxcr3l*, and *cxcr5* clades form a clade with a bootstrap value of 57% (Figure 4). L dominant expression of *cxcr3* was observed in the lung, intestine, kidney, and spleen with different expression (HCDE), whereas S dominant expression of *cxcr3l* was observed in the lung and spleen.

*ccr7* and its surrounding genes (*smarce1* and *tns4*) were conserved between human and *X. laevis*. Unfortunately, locus in *X. tropicalis* was not identified in available genome sequences. RNAseq analysis demonstrated expression in the spleen and dominant expression of *ccr7.L* in the intestine and *ccr7.S* in the testis (Figure 6). *CCR7* is a candidate receptor for *CCL19* and *CCL21* in human (Förster et al., 2008). Interestingly, dominant expression of *ccl21.S* in the intestine and *ccl19.L* and *ccl21.L* in the testis was observed. This inconsistent expression pattern of receptor and ligand in L *versus* S may serve as a model for crosstalk between subgenomes.

*ccr10* was L singleton and gene order around *Xenopus ccr10* was inconsistent with human. CCR10 binds to CCL27 in human (Homey et al., 2000). Slightly expression was observed in the heart and spleen.

*cxcr1* is a candidate for the receptor of the *cxcl8* cluster genes. Synteny was conserved in human adjacent to *arpc2* and *tns1,* although human ortholog was tandemly duplicated as *cxcr1* and *cxcr2* (Figure 2). RNAseq analysis demonstrated S dominant expression in embryogenesis and spleen, and transcriptome analysis indicated different expression (HCDE).

*cxcr4* and *ackr3* were receptors for *cxcl12. cxcr4* was located between *thsd8b* and *dars.* In *X. tropicalis*, *cops8* was translocated within *X. tropicalis* chromosome 9 (XTR9). Almost similar embryonic expressions of both *cxcr4.L* and *cxcr4.S* were detected from stage 9 (late blastula). In adult tissues, transcriptome correlation analysis indicated different expression (HCDE) as L dominant expression in the intestine, liver, heart, and spleen. *ackr3* was located between *iqca1* and *cops8*, and L-dominant expression was detected from stage 9. Almost L dominant expression was observed among adult tissues. However, S was dominant in testis. Transcriptome analysis indicated different expression (HCDE).

## Discussion

This study comprehensively identified and analyzed chemokine ligands and their receptors in *X. laevis* genome. L subgenome retained genes are dominant as 13 for L singleton genes *versus* one for S singleton of the identified genes, consistent with the S subgenome having a faster rate of pseudogenization than the L after allopolyploidization *Xenopus* species (Furman et al., 2018). Transcriptome correlation analysis suggests that the genes of 13 different expression (DE) homeologous pairs include potential candidates for subfunctionalization or neofunctionalization.

For the retention rates of homeologous gene pairs, all ligand genes in *X. laevis* genome showed 71% (22/31) in this study. This rate was higher than all analyzed genes (56%; 8,806/15,613) reported by Session et al. (2016). The details of that were 71% (10/14) for CXC-type, 71% (10/14) for CC-type, and 100% (2/2) for XC-type ligands; no significant differences between them was observed, suggesting that WGD promotes constant evolutionary divergence of ligands because it ensured diversity and increased the likelihood of acquiring novel functions such as antibacterial activity (Hieshima et al., 2003). This idea may be supported by transcriptome correlation analysis that revealed a higher rate of different expression and L or S dominant expression pairs in chemokine ligands (9 of DE *vs*. 2 of SE, one L-dominant and one S-dominant, Table 1).

The retention rate of all chemokine receptor homeologous pairs (53%, 9/17) was similar to all analyzed genes. However, details were 86% (6/7) for CXC-type (including *ackr3*), 29% (2/7) for CC-type (including *ackr4*), and 33% (1/3) for XC-type chemokine receptor. S subgenome gene loss of CC and XC may depend on “genome fractionation” (Schnable et al., 2011; Sankoff et al., 2012; Garsmeur et al., 2014). In contrast, the CXC-type receptors and the candidate CXC-type ligands tended to have higher retention rates, suggesting selective pressure for dosage compensation or subfunctionalization in their expression domain or target specificity (Session et al., 2016; Watanabe et al., 2017). As another example, the homeologous pairs of the ligands and receptors involved in growth factors showed the highest retention rate for TGF, FGF, and Wnt signaling (Michiue et al., 2017; Suzuki et al., 2017).

*dN/dS* analysis revealed four genes, *cxcl8b.1.S*, *cxcl18.S*, *ccl21.S*, and *xcl1.L*, had a *dN/dS* ratio greater than one, and four genes, *cxcr4.L*, *cxcr4.S*, *ackr3.L*, and *ackr3.S*, had shallow ratios of less than 0.1. Referring to ratios from automatically calculated results from Session et al. (2016), the homeologous genes with *dN/dS* ratios greater than one were only 0.3% [45 in 17,590 genes (8,795 homeologous pairs)] and less than 0.1 were 32% (5,561 in 17,590). These findings suggest a higher tendency of relaxation in the chemokine genes among homeologous genes.

Regarding *cxcl8* genes, the homeologs of *cxcl8a.1* and *cxcl8a.2* showed a high correlation (Table 1), and the pattern of the expressed organs was also similar. These genes possess ELR motifs and are predicted to promote the migration of neutrophils (Strieter et al., 1995). Since *cxcl8a.1* gene expression was upregulated by virus infection (Koubourli et al., 2018), it may function in the early response to infection and inflammation in *Xenopus*. In contrast, *cxc8b.2.L* recruited anti-inflammatory macrophages, which expressed genes associated with immune suppression (Koubourli et al., 2018). During inflammation and tissue repair, there is the recruitment of proinflammatory M1 macrophage, followed by anti-inflammatory M2 macrophage (Murray and Wynn, 2011). Diversified *cxcl8b* genes in *X. laevis* may play a different role in regeneration and tissue repair.

We found the expression of *ccr2* and *ccr8* in oogenesis, suggesting that these genes act in oogenesis or as a maternal factor. *ccr2* was also broadly expressed in adult tissues (Figure 6), reflecting its expression in macrophages and lymphocytes. Although a ligand for *ccr2* was not identified in *Xenopus*, *ccl42b*, *ccl42c*, and *ccl42d* conserved synteny and retained similarity with the *CCL2*, which is human *CCR2* ligand (Zlotnik and Yoshie, 2000). Therefore, some of these may be candidates for the ligand of *Xenopus ccr2*. Actually, not in the oocyte, but the weak expression of *ccl42b.S* was detected in the ovary (Figure 6). Next, regarding *ccr8*, among all the receptors examined in this study, only *ccr8.L* showed dominant expression in the testis and ovary. *CCL1* and *CCL18* were known as ligands for *CCR8* (Garlisi et al., 1999; Islam et al., 2013), but both have been unidentified in *Xenopus*. Interestingly, although *ccr2* and *ccr8* are not identified in teleosts (Table 1), *CCR2* and *CCR8* RNAs have also been detected in the human oocyte (Zhao et al., 2020). Although their role in oocytes is still unclear, both genes may have evolutionarily conserved functions.

*cxcl12*, *cxcr4*, and *ackr3* had been examined their expression and function in the early development of *X. laevis* (Moepps et al., 2000; Braun et al., 2002; Fukui et al., 2007; Takeuchi et al., 2010; Mishra et al., 2013; Shellard and Mayor, 2016). Because sequence homology of each ortholog is relatively well conserved among vertebrates (DeVries et al., 2006; Nomiyama et al., 2013), *cxcl12*, *cxcr4*, and *ackr3* are anticipated to undergo intense purifying selection. This prediction was also supported in this study. In contrast, the expression levels of *cxcl12.S* and *ackr3.S* were reduced compared to L counterparts in early development, and all three homeologous pairs indicated HCDE in adult tissues. These findings suggest that the potential subfunctionalization/pseudogenization is progressing in homeologs of *cxcl12*, *cxcr4*, and *ackr3*.

## Data Availability

The datasets presented in this study can be found in online repositories. The names of the repository/repositories and accession number(s) can be found in the article/Supplementary Material.
